# A microscope in your pocket: can smartphones be used to perform microsurgery?

**DOI:** 10.1590/acb392524

**Published:** 2024-05-24

**Authors:** Balduino Ferreira de Menezes, Lucas Vannuchi Magnani, Matheus Scuracchio Fernandes, Luis Fernando Spagnuolo Brunello, Thales Fernandes de Souza, Fausto Viterbo

**Affiliations:** 1 Universidade Estadual Paulista – Faculdade de Medicina – Botucatu (SP), Brazil.; 2 Autonomous Researcher – Botucatu (SP), Brazil.

**Keywords:** Anastomosis, Surgical, Microsurgery, Smartphone

## Abstract

**Purpose:**

To evaluate the use of the latest generation smartphone camera in performing arterial microanastomosis in rats.

**Methods:**

Ten Wistar rats were divided into 2 groups and underwent anastomosis of the right carotid artery with the aid of magnification from a microscope (group M) and a smartphone camera (group S), to compare patency in 72 hours, as well as to measure the weight of the animals, diameter of the carotid arteries and anastomosis time.

**Results:**

There was no statistical difference between the weight of the animals or the diameter of the carotid arteries. There was a statistical difference for the time spent on anastomoses, which was greater in group S, with higher rates of thrombosis (p < 0.05).

**Conclusions:**

Although our patency and anastomosis time results were statistically lower in the smartphone group, there was success in some cases. As the segment continues to progress, it is likely that the results will improve in line with the evolution of camera technology.

## Introduction

Microsurgery is a fundamental tool for the reconstructive plastic surgeon and requires a long and winding learning curve^1^.

Mechanisms to facilitate and enhance training are always welcome and can contribute to train the safe and more efficient practice of microsurgery in a clinical setting^2^.

Smartphones are rapidly improving in their graphical capacity and in the performance of variable tasks. Its use in plastic surgery has been established through several applications, from helping with information dissemination to better understanding patients’ desires^3^.

The idea of using the magnification of cell phone cameras was initially proposed by Kim et al.^4^ in 2015, using smartphones with less advanced technology up to devices with higher technology, mostly focused on home training.

Despite more recent attempts, others have been unable to achieve the same level of patency as achieved with a microscope^5^^,^ 6.

However, the enormous range of possibilities that the use of an instrument so widespread and present in people’s daily lives can take microsurgery to environments with fewer resources and encourage new surgeons to develop in the prestigious field of plastic surgery.

The objective of this study is to evaluate the possibility of performing microsurgical arterial anastomosis in live training models using one of the most popular smartphones of our time.

## Methods

The project was developed after approval by the Universidade Estadual Paulista – Botucatu ethics committee (number 1441/2023), based on the ethical principles of animal experimentation approved by the Brazilian College of Animal Experimentation, always considering the pain and discomfort of the animals involved in the study.

The research was carried out on 10 male Wistar rats (Rattus norvegicus), that were 120 days old. The animals were kept in cages designed for rats, ensuring their circadian rhythm was preserved, and a supply of water and food ad libitum.

The anesthesia used was Ketamine (Cetamin, Syntec, Brazil) in doses of 80mg/kg and Xylazine (Xilazin, Syntec, Brazil), 10mg/kg, intraperitoneally, and repeated as necessary. Before the end of the surgery, a dose of Tramadol (Tramadon, Cristália, Brazil) 5mg/kg was administered intramuscularly. After the procedure, a parenteral bolus of Dipirona Sodica (Febrax, Lema-Injex Biologic, Brazil) 500 mg/Kg was used subcutaneously, once a day for two days, according to the routine at the FMB-UNESP vivarium. After anesthesia, cervical trichotomy was performed on all animals.

**Figure 1:**
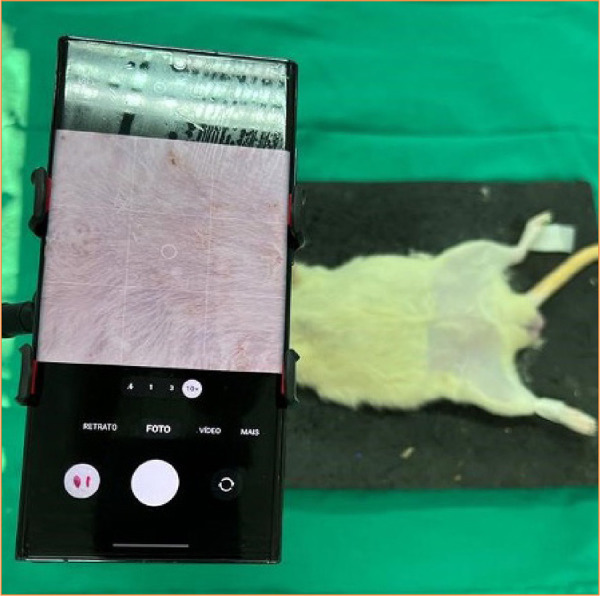
Demonstrative image of smartphone position.

**Figure 2:**
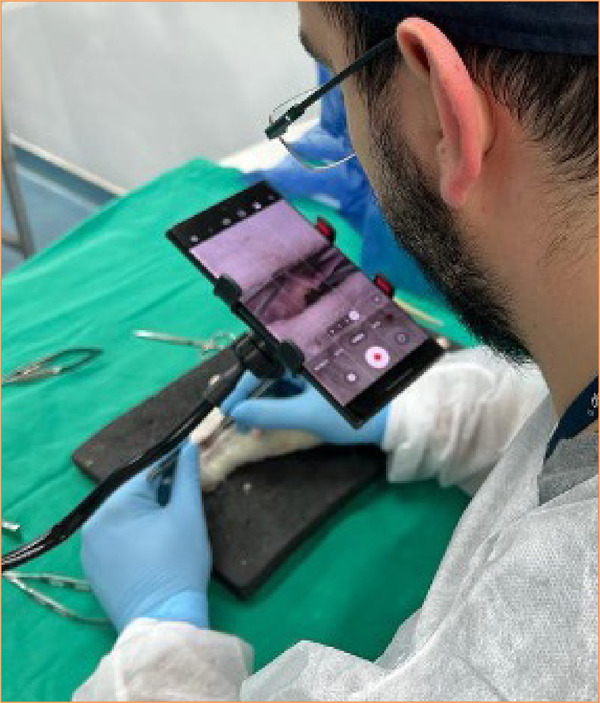
Demonstrative view of surgeon’s position during operation.

The animals were randomly divided into two groups of 5 animals each. In the first group, dissection and anastomosis were assisted by an optical microscope DF Vasconcelos, Brazil (group M). The second group underwent these procedures under the visualization of a smartphone (group S) model Samsung S23 Ultra^®^ (Fig. 1). The Smartphone was attached to the same support as the microscope, while an independent researcher manipulated the camera to increase or decrease the magnification level as directed by the surgeon (Fig. 2). The minimum magnification was 10 times, while the maximum was 14.5 times. As was observed in a previous experiment with a non-living model, the magnification of smartphones was unable to exceed 15 times due to image blurring and resolution loss. To assist with lighting, the surgeon used an auxiliary photophore equipped with a headrest (Dr-Kim, Korea).

Animals were kept in boxes of appropriate size, with only one animal in each box. The temperature was kept consistent (25 ± 2°C), and the 12-hour cycle was carefully observed. Water and food were provided ad libitum until euthanasia was performed. Aiming at environmental enrichment, tiny plastic balls and crumpled paper were offered, to provide tactile stimulation and alleviate stress in the animals, while still allowing the rats to move around in the box. Furthermore, the animals’ bedding was made of wood shavings, composed of large granules, ensuring the absorption of the animals’ urine.

The carotid artery diameters were assessed in both groups using a millimeter ruler and a 10x magnifying tool such as a microscope or smartphone.

The total duration of the anastomosis procedure was initiated when the artery was severed, and continued with single stitch placements. Ten animals underwent surgery by the same researcher (BFMN) and received arterial anastomosis using 10-0 mononylon and simple stitches. After the procedure, the animals were observed for 72 hours. Following this period, the rats underwent a different anesthetic procedure to check the vascular patency of the anastomosis. They were subsequently euthanized with as intravenous injection of 120mg/kg of intravenous Thiopental (Thiopentax, Cristália, Brazil), and their frozen carcasses were saved for training purposes. Patency was verified by the technique previously described^7^ using two forceps placed distally to the anastomosis, and a maneuver was made to empty the central region at both sites. Subsequently, the proximal forceps were released to refill the central space. If the created intravascular space was immediately filled with blood in the proximal to distal direction, we deemed it a to be successful patency (flow). Vessel staining and pulsation were considered indirect signs of successful patency (flow).

**Figure 3:**
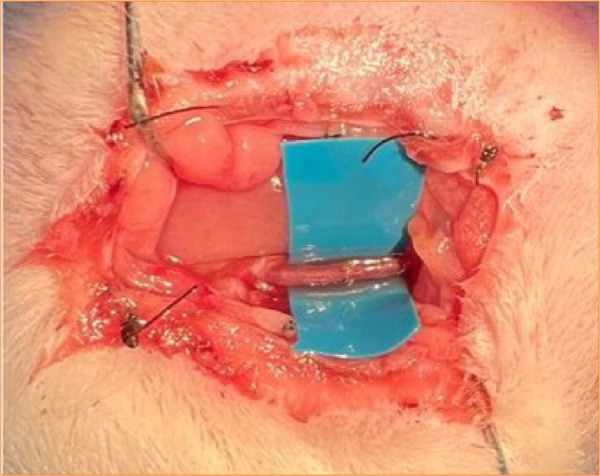
72 hour patency showing good color and flow after the procedure.

All surgeries were conducted by the same researcher who had previous training in experimental microsurgery, but had not previously used a smartphone as a microscope in live models. Data obtained were analyzed using OpenEpi software, version 3. For the variables of animal weight, artery diameter and anastomosis time, Mann-Whitney U test was used; while for the patency variable, Fisher Exact test was used (Fig. 3).

## Results

The study was carried out in May 2023, at the Experimental Research Unit of Botucatu Faculty of Medicine. All surgeries were performed by the first author, while patency and measurements were checked out by an independent researcher (LVM).

The microscope group (M) utilized magnifications of 10, 16 and 25 times in their procedures, while the smartphone group (S) recorded videos at magnifications of 10, 12.5 and 14.5 times.

Average animals weight was 227.8g in group M and 256g in group S (p = 0.2492), while the average diameter of the arteries in group M and group S was 1.16mm and 1.2mm respectively (p = 0.4762).

The Mann-Whitney U test was utilized to compare the differences between the microscope and smartphone groups in microsurgery training. This non-parametric test revealed a significant disparity in anastomosis times. By utilizing a microscope, the task was finalized in an average time of around 26 minutes, a marked difference from the 48 minutes it took for individuals using a smartphone. (p < 0.05). This emphasizes the efficiency gap between the two methods.

For variables such as initial weight of subjects and artery diameter, the Mann-Whitney U test indicated no significant differences (p > 0.05), suggesting consistency in these factors in both training methods. Furthermore, the patency rates after 30 minutes, analyzed using the same test, showed no significant difference, implying that the effectiveness of microsurgery was similar regardless of the training tool employed.

In the microscope group, the patency rate within 72 hours was 100%, significantly higher than the 40% in the smartphone group. Results are seen in Table 1:

**Table 1:** Comparison of weight, right carotid artery diameter, time of anastomosis and 72-hour patency.

Group M - Microscope				
Rat	Weight (grams)	Right Carotid Artery Diameter (milimeters)	Time (minutes)	72-hour Patency
1	194	1.1	25	Yes
2	210	1.1	26	Yes
5	224	1.2	28	Yes
7	286	1.3	26	Yes
9	225	1.1	26	Yes
**Group M - Microscope**				
**Rat**	**Weight (grams)**	**Right Carotid Artery Diameter (milimeters)**	**Time (minutes)**	**72-hour Patency**
3	200	1.1	47	No
4	218	1.1	44	No
6	270	1.2	49	No
8	300	1.3	51	Yes
10	292	1.3	49	Yes

## Discussion

The widespread use of cell phones has had a noticeable effect on the daily lives of entire population, including doctors from different specialties. Plastic surgeons are no exception, with approximately 90% of them regularly incorporating this device in their routine^8^.

Various applications and uses for smartphones are multiplying, however, in the field of microsurgery, only a few experiences were published to date^2^^,^ 4 ^,^ 9.

Since microsurgery is an area with a long learning curve and emphasis on training for optimal clinical application^10^, the field of microsurgery requires innovative tools to enhance our practice^11^.

Microsurgery training using cell phones was first described by Kim et al. in 2015 and later developed by other authors using better technologies^12^^,^ 13. It was carried out using inert materials and obtained encouraging results. Subsequently, Thibault et al.^14^ carried out research aiming to validate the effectiveness of cell phones as a training tool for residents, specifically in performing end-to-end anastomoses and subsequently carrying out a self-evaluation.

In 2019, Teixeira et. al.^15^ attempted to perform an in vivo study evaluating the patency of the arterial anastomosis in live rats, but were unable to complete any surgery due to the incapacity of the technology at the time.

Animal models play a vital role in the training of microsurgeons as they provide the most realistic approach to mastering anastomoses^16^^,^ 17.

Therefore, with the constant evolution of modern smartphones and the effectiveness obtained using cell phones as a training model, it was decided to conduct an in vivo study to assess the effectiveness of the surgical procedure as an experimental model under the hands of an experienced microsurgeon, while adhering to the principles of the 3Rs^18^.

Patency rates in experimental microsurgery vary greatly in the literature and depend on training time, experience, size of the vessels addressed and observation time of the animals to assess patency^7^. Therefore, the result of a high patency rate in the microscope group was expected, suggesting that the smartphone group should also achieve a success rate of at least 50%.

It is probable that the low resolution in group S did not allow a proper elimination of the adventitial layer, potentially contributing to the development of vascular thrombosis within a 72 hour period. As cameras continue to advance, there is a possibility that this type of suturing in vessels smaller that 1mm become achievable. The 1.3mm vessels were successful in creating the anastomosis in both cases, suggesting that in larger vessels it would be possible to have achieved similar patency when viewed under a microscope. we

Although the smartphone used has a magnification of up to 100x, a maximum approximation of 14.5x was used, since correct focus cannot be achieved with higher magnifications. Furthermore, a challenge faced was the reduction in three-dimensional imaging, which is most effective when using a microscope, posing difficulties in carrying out microsurgical procedures.

Currently, the development of technologies that allow the viewing of virtual footage through VR14 glasses is growing exponentially. Hence, in the coming years it will be possible to merge these emerging technologies in microsurgical training and practice, providing an unparalleled experience for surgeons.

One limitation of this study is that it did not include venous anastomoses; However, in general, these stitches are more complex and require a more delicate handling material, which was not available in our laboratory. Moreover, there is no need for a designated support in upcoming clinical applications, as the current generation cell phones are light and can be attached to headrest devices.to . This is comparable to photophores or other tools that can be attached to the patient or the surgical table, and can be sterilized or covered with sterile plastic.

## Conclusion

Despite the lower patency and anastomosis time results in the smartphone group, the constant evolution of the segment suggests that in the future the results obtained may be better in line with the evolving technology.

## Data Availability

Data sharing is not applicable.
